# A Novel Method for the Diagnosis of Transverse Maxillary Deficiencies Based on CBCT

**DOI:** 10.3390/diagnostics16071034

**Published:** 2026-03-30

**Authors:** Daniel Diez-Rodrigálvarez, Elena Bonilla-Morente, Alberto-José López-Jiménez

**Affiliations:** 1 Postgraduate Program in Orthodontic and Dentofacial Orthopedics, Institución Universitaria Mississippi, 28010 Madrid, Spain; daniel.diez@dentactil.es (D.D.-R.); eleboni@yahoo.com (E.B.-M.); 2 Deparment of Ortodoncia, Department of Biotechnology, Medicine, and Biosanitary Sciences, Francisco de Vitoria University, 28223 Madrid, Spain; 3 Department of Dental Clinical Specialties, Complutense University of Madrid, 28040 Madrid, Spain

**Keywords:** cone-beam computed tomography, transverse diagnosis, maxillary transverse deficiency, buccolingual inclination, molar angulation, dentoalveolar compensation, skeletal discrepancy, orthodontic diagnosis

## Abstract

**Background/Objectives:** To Develop a CBCT-based transverse diagnostic method that establishes normative buccolingual inclination values for permanent first molars and objectively distinguishes between dentoalveolar transverse deficiency and skeletal maxillary deficiency. **Methods**: A total of 1120 initial CBCT scans were reviewed, and 40 subjects with normal occlusion met the inclusion criteria. Volumes were reoriented using a standardized three-plane protocol, and molar angulations were measured relative to reference planes parallel to the occlusal plane. Intra- and inter-examiner reliability were assessed using ICC. Descriptive, comparative, and correlation analyses were performed bilaterally and between arches. **Results**: No significant right–left differences were observed for upper molar angulation (URM vs. ULM: 99.5° vs. 99.1°; t(19) = 1.560, *p* = 0.135) or lower molar angulation (LRM vs. LLM: 78.9° vs. 78.9°; t(19) = 0.301, *p* = 0.767). Non-parametric analysis confirmed these findings (ULM vs. URM: Z = −1.203, *p* = 0.229; LLM vs. LRM: Z = −0.427, *p* = 0.669). Significant positive bilateral correlations were observed in both arches (upper: r_S_ = 0.784, *p* < 0.001; lower: r_S_ = 0.837, *p* < 0.001). A significant negative correlation was found between upper and lower molar angulations (left side: r_S_ = −0.626, *p* = 0.003; right side: r_S_ = −0.858, *p* < 0.001), demonstrating dentoalveolar compensation. **Conclusions**: CBCT enables the precise assessment of molar buccolingual inclination and the establishment of normative patterns essential for transverse diagnosis. The proposed method allows the quantification of the maxillary “basal defect” after virtual dental decompensation, providing an objective tool to differentiate dentoalveolar from skeletal transverse discrepancies and guide targeted treatment planning.

## 1. Introduction

Traditionally, the diagnosis of transverse maxillary discrepancies—differences in width between upper and lower jaws—relies on clinical assessment. Posterior crossbite (when upper teeth bite inside the lower back teeth), whether unilateral or bilateral, and crowding remain the main factors for deciding on expansion. However, this clinical approach often fails to identify the origin of the deficiency. It does not clarify whether the cause is dentoalveolar (teeth and supporting bone), skeletal (jawbone), or both.

The difficulty in making this distinction—i.e., distinguishing between different types of malocclusion or misalignment—has made transverse diagnosis—a process for evaluating jaw width and asymmetry—a challenge in modern orthodontics. Cephalometrics, the measurement of the head using lateral (side-view) skull radiographs, are effective for sagittal (front-to-back) and vertical (up-and-down) analysis. However, the frontal (posteroanterior, or PA) projection has been unreliable for transverse (side-to-side) evaluation [1,2]. The limitations of this technique include superposition, when structures overlap in the image, and a greater likelihood of patient positional errors. These issues have reduced the precision of conventional anteroposterior cephalometric analyses, which are assessments performed on front-to-back images of the skull.

As a result, the main tools used for the transverse plane have been study models (Wilson curve analysis, which assesses dental arch curvature relative to molar position, and occlusograms, diagrams of occlusal relationships) and occlusal photography (e.g., Wala Ridge, the bony ridge near the mandibular basal bone) [3,4,5,6]. The introduction of Cone-Beam Computed Tomography (CBCT), a 3D imaging technique for detailed views of dental and skeletal structures, has transformed diagnosis. This technology provides a distortion- and superposition-free frontal view. It provides the three-dimensional information needed to accurately determine the extent of patient dentoalveolar (tooth and alveolar bone) and skeletal transverse problems [7,8,9]. Many authors have adopted CBCT for its measurement accuracy and lack of superimpositions, enabling a new era in diagnosis.

Therefore, the objective of this study was twofold. First, we aimed to develop a CBCT-based transverse analysis using normative reference values for the inclinations of permanent first molars. Second, we sought to design and implement a diagnostic method. This method objectively differentiates dentoalveolar from skeletal transverse problems and helps plan specific treatments for each component.

## 2. Materials and Methods

### 2.1. Sample Determination

The present study is a descriptive design aimed at establishing the normative values of the transverse inclination of permanent first molars in a population with normal occlusal alignment. The study was conducted at the Mississippi Institution University Clinic and Complutense University of Madrid.

A total of 1120 initial CBCT files from the clinic’s database were analyzed. Only files obtained for non-orthodontic diagnostic reasons (such as dentoalveolar pathology evaluation or implant planning) were included.

For the sample size calculation, a total population of 1120 patients was considered, with a 90% confidence level and a 10% margin of error. Assuming an expected proportion of success or characteristic of interest of 50% (*p* = 0.5), representing the most conservative scenario, the finite population correction was applied.

The calculation yielded an optimal sample size of 62 participants, a number that ensures a representative estimate of the total population under the established statistical parameters. Therefore, a sample of approximately 60 to 65 patients was considered sufficient to achieve the desired precision and confidence level in the present study. It is worth noting that the mean sample size in previous studies ranged between 30 and 80 patients per study.

After applying the eligibility criteria, the final sample consisted of 40 patients. Thirty files were discarded due to artifacts or acquisition errors preventing proper landmark identification. This sample size was considered suitable and was comparable to reference studies on transverse norms based on CBCT, which generally include 30 to 80 subjects.

All patients who met the inclusion criteria were consecutively included between 2019 and 2024.

The mean age of the sample was 18.6 years. The youngest patient was 17.3 years old, and the oldest was 21.4 years old. There were 26 women and 14 men. All 40 scans were performed at the same radiological center using the same machine (I-CAT scanner, Alpharetta, GA, USA; 0.4 mm voxel size; 20 s CBCT acquisition). The CBCT analysis was performed using Dolphin imaging 12 (v12.0) (Chatsworth, CA, USA).

### 2.2. Eligibility Criteria

The inclusion criteria were as follows. Patients must have completed growth (no further height increase expected) and be of Caucasian origin. They needed a complete permanent dentition (all adult teeth present) and a skeletal Class I (jaw relationship considered normal) based on the ANB angle (angle between points A, Nasion, and B on a cephalometric radiograph) and Wits appraisal (measurement of jaw position on a lateral skull radiograph). Study models had to meet Andrews’ six keys to occlusion (six characteristics used to identify ideal dental alignment) to indicate normal occlusion. Patients should not have had previous orthodontic treatment.

The exclusion criteria were as follows. Patients with facial or skeletal asymmetry, TMD problems, or craniofacial anomalies were excluded. Patients with supernumerary teeth, agenesis, or prematurely lost teeth were not considered. Those with previous orthodontic treatment were also excluded.

### 2.3. Ethical Considerations

The study was conducted in accordance with the Declaration of Helsinki and approved by the Institutional Review Board (Ethics Committee) of Universidad Complutense de Madrid. The initial ethical approval was obtained in June 2025 due to the loss of the previous ethics approval documentation. A new ethics approval was obtained (protocol code 607_CE_20260212_14_SAL and date of approval 12 February 2026). Written informed consent was obtained from all participants for the use of their CBCT images for diagnostic purposes. The CBCT files were preexisting and anonymized prior to inclusion, in compliance with current data protection laws.

### 2.4. CBCT Orientation and Measurement

**Figure 1:**
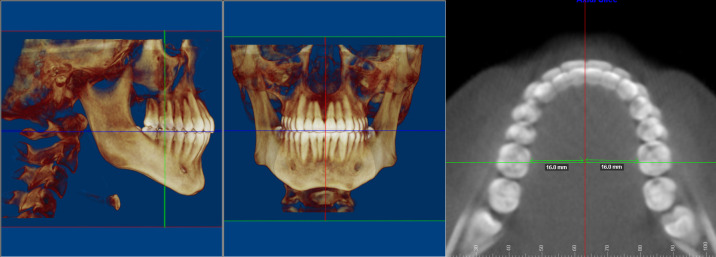
Obtaining the front section for transverse measurement.

To obtain the same frontal slice in all patients, the CBCT volume was reoriented using the three-dimensional standardization method proposed by Balachandran R et al. [10]. The following reference planes were established for transverse measurement (Figure 1):

1. Sagittal Plane: Established by passing through the midline of the central teeth and rotating the volume until the sagittal axis is equidistant from the palatal surface of the first molars.
2. Axial Plane: Located at the height of the occlusal surface of the lower first molars.
3. Coronal Plane: Placed at the center of the main fossa of the lower first molars.

**Figure 2:**
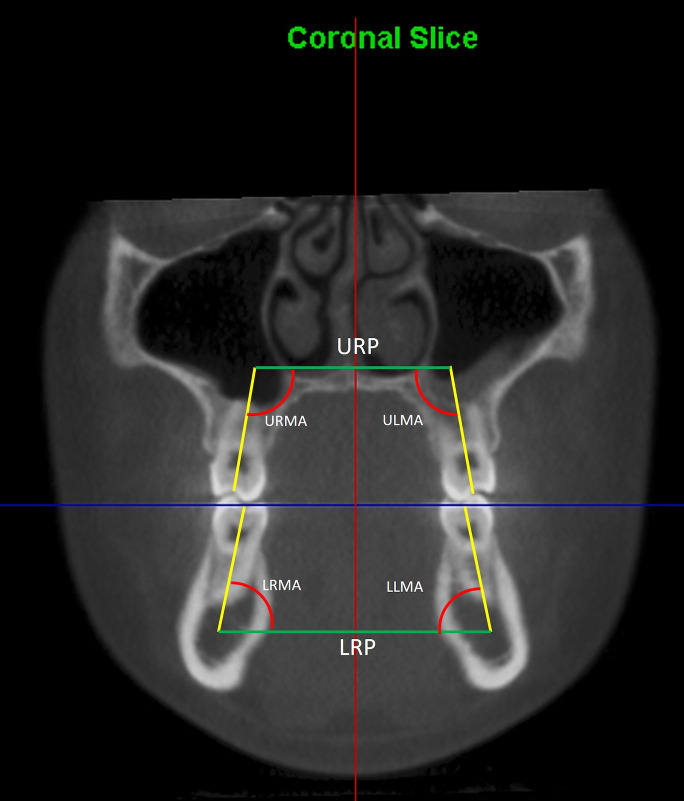
Angulations of permanent molars for transverse analysis. Green line, URP: upper reference plane; LRP: lower reference plane. Yellow line, axis of length of the first molar. Red curves: angulations with the URMA, ULMA, LRMA, and LLMA planes.

Once the frontal slice was set, reference planes and angles were defined (Figure 2):

• Occlusal Plane (OP): Defined as the line passing through the contact points between the palatal cusp of the upper molar and the main fossa of the lower molar. It runs parallel to the horizontal reference used for correct coronal orientation of the CBCT.
• Upper Reference Plane (URP): This is a line parallel to the occlusal plane (OP) and is positioned above the apices of the upper molars.
• Lower Reference Plane (LRP): This is a line parallel to the occlusal plane (OP) and is positioned below the apices of the lower molars.
• Longitudinal Axis: Defined as the line running from the central or main fossa of the occlusal surface to the root center at the apical level in the coronal slice.

Finally, inclination angles (Upper Rigth Molar Angle -URMA-, Upper Left Molar Angle -ULMA-, Lower Rigth Molar Angle -LRMA-, Lower Left Molar Angle -LLMA-) were measured between the longitudinal axes and their respective reference planes (URP and LRP).

## 3. Statistical Analysis

Data were collected in an Excel 2007 database, and the SPSS version 29.0 program was used for statistical analysis.

### 3.1. Evaluation of Reliability (Measurement Error)

The reliability of the measurements (both intra- and inter-examiner) was checked using the Intraclass Correlation Coefficient (ICC). The main examiner (DDR) traced the planes and angles twice, with a 2-week gap, to calculate intra-examiner reliability. A second calibrated examiner measured a random subsample (10 scans) to check inter-examiner reliability, with a Kappa index of 0.812, indicating a high intra-examiner reliability.

### 3.2. Descriptive and Comparative Analysis

For each angular variable, descriptive statistics were calculated. This included the mean, standard deviation, range, and 95% confidence interval. Data normality was tested with the Shapiro–Wilk test.

• Parametric variables (right vs. left side angulation) were compared using Student’s *t*-test for paired samples.
• For nonparametric distributions, the Wilcoxon test was used for comparisons.

### 3.3. Correlation

Finally, the Spearman correlation coefficient (r_S_, a non-parametric measure of statistical association) was used to evaluate the correlation between the angulations of the right and left sides (symmetry) and between the variables of the upper and lower arches. The level of statistical significance (probability threshold for meaningful results) was set at *p* < 0.05.

## 4. Results

The results for the values obtained from the measurement of the angles between the upper and lower reference planes and the long axis of the molars are shown in the following table (Table 1).

## 5. Parametric Analysis

• Comparing ULM vs. URM, no statistically significant differences were found between the right-side and left-side measurements; t(19) = 1.560, *p* = 0.135. Mean ULM: 99.1; Mean URM: 99.5.
• Comparing LLM vs. LRM, no statistically significant differences were found between the right-side and left-side measurements; t(19) = 0.301, *p* = 0.767. Mean LLM: 78.9; Mean LRM: 78.9.

## 6. Non-Parametric Analysis

• Comparing ULM vs. URM, no statistically significant difference was found between right- and left-side measurements (Z = −1.203, *p* = 0.229). Median ULM: 99.5; Median URM: 99.5.
• Comparing LLM vs. LRM, no statistically significant differences were found between the right-side and left-side measurements; Z = −0.427, *p* = 0.669. Median LLM: 79.5; Median LRM: 80.0.

**Figure 3:**
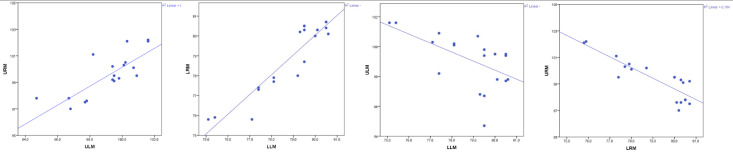
Correlation of permanent molar measurements.

Correlation between Right and Left Sides and Upper and Lower Arches ( Figure 3 ):

We observed the relationship between variables using the Spearman correlation coefficient to determine whether the variables behaved similarly bilaterally and, crucially, whether there was a correlation between upper and lower changes.

• Upper: ULM and URM: The relationship is positive and significant; r_S_ = 0.784, *p* < 0.001. As ULM scores increase, URM scores also increase.
• Lower: LLM and LRM: The relationship is positive and significant; r_S_ = 0.837, *p* < 0.001. As LLM scores increase, LRM scores also increase.
• Left: ULM and LLM: The relationship is negative and significant; r_S_ = −0.626, *p* = 0.003. As ULM scores increase, LLM measures decrease.
• Right: URM and LRM: The relationship is negative and significant; r_S_ = −0.858, *p* < 0.001. As URM scores increase, LRM measures decrease.

## 7. Discussion

Unlike vertical and anteroposterior discrepancies, the diagnosis of transverse maxillary deficiencies (TMD) has been extremely difficult until now [11,12,13,14]. Clinical evaluation, model analysis, occlusogram, and radiographic measurements have been used in conjunction or separately to aid in correct diagnosis. But only the advent of CBCT has enabled a true anteroposterior projection of molar position and its relationship to the skeletal base. Many authors have used CBCT to address the transverse problem in new ways [15,16,17,18,19,20]. However, collectively, they offer disconnected information.

Some analyze molar relationships in only one jaw, either the lower [19] or the upper [20]. Others use CBCT to study the skeletal and dentoalveolar effects of treatments, such as SARPE with different expanders [21,22,23], unilateral disjunction [18], and the relationship between molars and pattern [19]. Only two articles have proposed a study seeking normative values. Tong et al. [24] conducted a study on the angulations in normal inclinations of each tooth. Their reference plane is vertical, not horizontal, but if we translate the angles they obtain to the occlusal plane reference, their angulations are compatible with those obtained in our study. They also define the tooth’s long axis in the same way. However, this article is merely descriptive and does not relate the obtained values to the transverse maxillary problem. Miner et al. [18] used angular and linear measurements to define normality in healthy patients, but no correlation was observed between the chosen angle (the long axis of the tooth-occlusal plane) and the obtained values. Furthermore, the use of skeletal reference planes (the mandibular base and a tangent to the nasal fossa base) can artificially alter values due to anatomical variability among patients.

The strong positive bilateral correlations observed in both arches (r_S_ = 0.784 in the maxilla and r_S_ = 0.837 in the mandible) indicate a high degree of transverse symmetry in subjects with normal occlusion. From a clinical perspective, this finding establishes that symmetrical molar angulation represents a normative pattern. Therefore, clinically relevant unilateral deviations from these values may indicate transverse imbalance or asymmetric dentoalveolar compensation, even in the absence of evident posterior crossbite.

More importantly, the statistically significant negative correlations between upper and lower molar angulations (r_S_ = −0.626 on the left and r_S_ = −0.858 on the right) provide quantitative evidence of a natural dentoalveolar compensatory mechanism. Clinically, this means that, when a skeletal transverse discrepancy exists, the dentoalveolar structures may adapt by increasing the buccal inclination of the maxillary molars and/or lingual inclination of the mandibular molars in order to maintain functional occlusion. This compensation can mask the true magnitude of the skeletal maxillary deficiency during routine clinical examination or when relying exclusively on intermolar width measurements.

Thus, the statistical significance in our study translates directly into diagnostic relevance. The identification of normative angulation values enables virtual dental decompensation, allowing clinicians to isolate the skeletal component of the transverse discrepancy. In practical terms, this distinction is essential for treatment planning, as dentoalveolar compression requires orthodontic correction, whereas a true skeletal basal defect may indicate the need for orthopedic or surgically assisted maxillary expansion. By converting angular differences into linear millimetric objectives, the proposed method transforms statistical findings into a reproducible and clinically applicable diagnostic protocol.

## 8. Clinical Application

The objective of the study is not merely to describe normality, but to seek a “method” for its clinical application in the diagnosis and treatment planning of transverse problems. The results demonstrate a negative correlation between upper and lower molar inclinations in normal individuals and provide objective evidence of a natural dentoalveolar compensation. It is precisely this compensation that masks the true skeletal discrepancy, making an analysis that virtually decompensates the dental position essential to identify the basal deficit. For this, we base the method on two basic principles:

1. Our appliances and treatments produce a vestibulolingual inclination of the molars without significantly displacing their apices. This movement results in an increase or decrease in the intermolar distance in the occlusal plane of 1 mm for every 3°, for an average molar length of 19.85 mm [9,10,11,12].
2. With the exception of specifically diagnosed cases requiring SARME (Surgically Assisted Rapid Mandibular Expansion), we do not treat defects of the mandibular basal bone [13].

**Figure 4:**
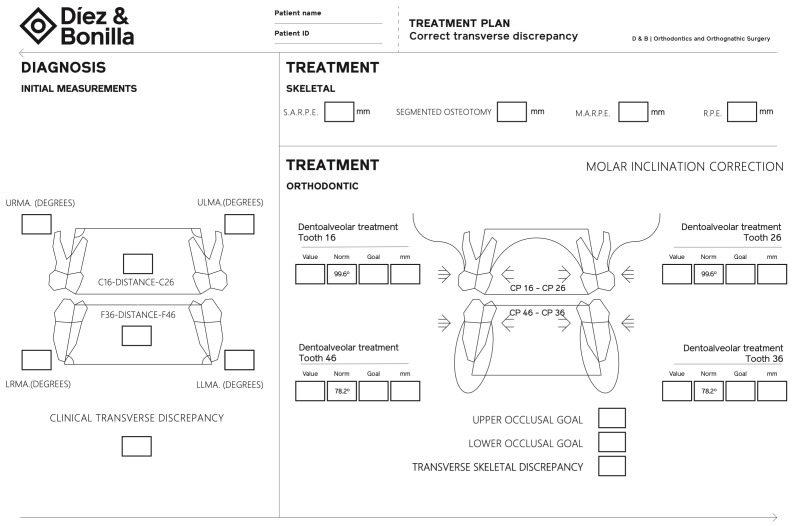
Diagnosis diagram of the transverse analysis.

Thus, the measurement of the distance between the main fossae of the lower first molars and the palatal cusps of the upper first molars will be the principal transverse clinical reference of the system described below (Figure 4):

1. Measure the distance between the main fossae of the lower first molars.
2. Measure the angles formed by the lower molars with the LRS and calculate the difference in degrees with respect to the norm on both sides.
3. Add or subtract the mm resulting from moving the molars to their correct angulation, at a rate of 1 mm linear for every 3 degrees of correction [12].

The result is the lower occlusal objective.

1. Measure the distance between the palatal cusps of the upper first molars.
2. Measure the angles formed by the upper molars with the URS and calculate the difference in degrees with respect to the norm on both sides.
3. Add or subtract the mm resulting from moving the molars to their correct angulation at a rate of 1 mm linear for every 3 degrees of correction.

The result is the upper occlusal objective.

1. The difference between the lower occlusal objective and the upper occlusal objective is the “basal defect” or skeletal deficiency of the maxilla.

Conceptually, this system is identical to the one we use for diagnosing anteroposterior problems. Once the lateral cephalometry tracing is obtained, we position the upper incisor according to the norm relative to its maxillary bone base. We position the lower incisor relative to the mandibular bone base. With both in their correct position, if there is an altered anteroposterior relationship between the two, it can only be of skeletal origin.

We have simply sought a standard of normality, as is done for other pathologies. Only if we know the norm can we diagnose the pathology, either by excess or by defect, and then apply the appropriate treatment. Since the beginning, we have felt the need to differentiate between transverse maxillary deficiencies that are dentoalveolar or skeletal, as dentoalveolar compression requires dentoalveolar treatment and a skeletal problem requires skeletal treatment. Lorente (2002) [25] classified and divided crossbites into skeletal and dentoalveolar types, clearly defining the need for such a differential diagnosis to apply the appropriate treatment in each case. Despite his enormous contribution, it was only a conceptual and descriptive classification, without associated measurements or defined normative patterns.

The fundamental advantage of our method is that it accurately locates the problem. But once the analysis is completed, no radiological follow-up is necessary, since our reference is occlusal. We can measure the interfossa distance of the lower molars and the distance between the palatal cusps of the upper molars at each appointment and regulate our treatments until the objective set in the initial analysis and treatment plan is achieved (Figure 4).

The study sample may seem small, but this study began in 2009, a time when the indication for CBCT was ethically debated due to radiation concerns. However, our results correlate with the angulations defined by Andrews in his studies to design the Straight Wire Appliance [4,23,24,25] and with Okeson’s studies [26] on the functional anatomy of occlusion, which define the ideal angulation of the molars for the cusps and main fossae to occlude correctly and allow the proper functioning of mandibular kinematics in the three axes of space. Likewise, the work of Tong et al. [24], previously mentioned, defines normality within an angulation range that is superimposable on ours.

Currently, the dilemma of whether CBCT is the imaging technique of choice for orthodontic diagnosis has been resolved. Larson B E [27] states that, although we still have much to learn about the best way to use CBCT, we know enough to consider it the imaging of choice for orthodontic treatment. The American Academy of Oral and Maxillofacial Radiology supports the safe use of CBCT in dentistry [28]. Although it is not a routine technique in children [29], its use is not contraindicated. The clinical evaluation of the patient will allow for individualized indication in each case. The most common indications for CBCT in children and adolescents are the location of impacted teeth in the premaxilla and the detection of root resorption [30]. From our perspective, the suspicion of a transverse problem should be included in the list of indications.

## 9. Conclusions

1. Cone-Beam Computed Tomography (CBCT) allows the precise assessment of the buccolingual inclination of the first molars, making it a fundamental tool for differential diagnosis between dentoalveolar transverse deficiency and the skeletal deficit (basal defect) of the maxilla.
2. CBCT is an increasingly safe technique that, following a rigorous clinical examination and judgment, can and should be indicated for patients.
3. Transverse problems should be included among the criteria for patient inclusion when recommending a CBCT.
4. Although studies with larger samples may refine the normative angular values, the reasoning process and conceptual validity of the proposed method remain unaltered, offering a precise protocol for treatment planning.

## Figures and Tables

**Table 1:** Measurements of molar angulation between reference plane and long axis of first molar (URMA: upper right molar angle; ULMA: upper left molar angle; LRMA: lower right molar angle; LLMA: lower left molar angle).

	Mean	Std. Deviation	Minimun	Maximun	95% Confidendence	
					Lower Bound	Upper Bound
URMA	99.500	1.6125	97.000	102.2	98.745	100.255
ULMA	99.110	1.7720	94.700	101.6	98.281	99.939
LRMA	78.910	1.7717	75.8	80.7	78.081	79.739
LLMA	78.865	1.6512	75.1	80.6	78.092	79.638

## Data Availability

The original contributions presented in this study are included in the article. Further inquiries can be directed to the corresponding author.
